# Targeted MRSA Decolonization Protocol to Reduce Surgical Site Infections in Implant-Based Breast Surgery

**DOI:** 10.1093/asjof/ojaf018.005

**Published:** 2025-05-13

**Authors:** Andrea Castaldo, Natalie Godfrey, Anam Furrukh, Anna Bogursky, Marcus McKenzie, John Castle

**Affiliations:** UMASS, Worcester, MA; UMASS, Worcester, MA; UMASS, Worcester, MA; UMASS, Worcester, MA; UMASS, Worcester, MA; UMASS, Worcester, MA

## Abstract

**Goals/Purpose:**

Surgical site infections (SSI) in implant-based breast reconstruction (IBBR) have been reported to occur with an incidence of 1-35%. These infections can often be treated with antibiotics but may also result in mental and physical distress, prolonged hospitalization, delayed adjuvant therapy, and failed reconstruction. Staphylococcus aureus (SA) is the most commonly implicated organism, and a correlation between SSI and methicillin-sensitive (MSSA) as well as methicillin-resistant (MRSA) Staphylococcus aureus colonization has been established. Some institutions have started to adopt standardized perioperative protocols, which include MRSA decolonization, to reduce SSI. However, literature has shown that universal MRSA decolonization protocols can have adverse effects, including high rates of mupirocin-resistant MRSA. This is the first study to test the effectiveness of targeted, rather than universal, MRSA decolonization in the reduction of IBBR SSI.

**Methods/Technique:**

The study used a single-center hybrid approach, combining retrospective and prospective data collection. For the retrospective component, 267 patients who underwent IBBR from 2010-2016 were studied. Data collected included surgical procedures, demographics, risk factors, and treatment of any infections, specifically antibiotics, rehospitalization, and reoperation. After establishing historical control data, an effort was made to decrease SSI through the implementation of a MRSA decolonization protocol. In this prospective component, 146 patients who underwent IBBR from 2017-2024 were studied. Preoperative MSSA/MRSA nasal and oral swab testing was performed, and if MSSA or MRSA positive, patients were enrolled in a MRSA decolonization protocol under the guidance of the Infectious Disease Department. This protocol included decolonization with five days of preoperative twice daily nasal mupirocin ointment, five days of preoperative chlorhexidine body washes, night-before and morning-of chlorhexidine oral rinses, and single-dose IV vancomycin administration 1 hour prior to surgery.

**Results/Complications:**

Overall infection rate was significantly lower in the protocol group as compared to the control group (19.2% versus 33.0%; P<0.003). When stratifying data by type of reconstruction, infection rate with expander reconstruction was also significantly lower in the protocol group as compared to the control group (16.7% versus 33.0%; P<0.05). No statistical difference in rate of infection was observed between the protocol and control group in regards to DTI reconstruction. Addition of neoadjuvant and/or adjuvant radiation or chemotherapy showed no statistically significant effect on incidence of SSI.

**Conclusion:**

The implementation of a targeted MRSA decolonization protocol in IBBR significantly reduces the rate of SSI. This reduction is especially evident in expander reconstruction and with more study may become evident in other forms of implant-based reconstructive and aesthetic surgery.

